# The association of erythropoietin-stimulating agents and increased risk for AV-fistula dysfunction in hemodialysis patients. A retrospective analysis

**DOI:** 10.1186/s12882-020-02209-6

**Published:** 2021-01-18

**Authors:** Anna Wärme, Henrik Hadimeri, Salmir Nasic, Bernd Stegmayr

**Affiliations:** 1 Dept of Internal Medicine and Clinical Nutrition, Institution of Medicine, University of Gothenburg, Gothenburg, Sweden; 2 Department of Nephrology, Skaraborg hospital, 541 85 Skovde, Sweden; 3 Research and Development Centre, Skaraborg Hospital, Skovde, Sweden; 4 Dept of Public Health and Clinical Medicine, Umea University, Umea, Sweden

**Keywords:** Arterio-venous fistula, Stenosis, Erythropoietin, Hemodialysis

## Abstract

**Background:**

Patients in maintenance hemodialysis (HD) need a patent vascular access for optimal treatment. The recommended first choice is a native arteriovenous fistula (AVF). Complications of AVF are frequent and include thrombosis, stenosis and infections leading to worsening of dialysis efficacy. Some known risk factors are age, gender and the presence of diabetes mellitus. The aim was to investigate if further risk variables are associated with dysfunctional AVF.

**Methods:**

This retrospective observational study included 153 chronic HD patients (Cases) referred to a total of 473 radiological investigations due to clinically suspected complications of their native AVF. Another group of chronic HD patients (*n* = 52) who had a native AVF but were without history of previous complications for at least 2 years were controls. Statistical analyses included ANOVA, logistic regression, parametric and non-parametric methods such as Student’s T-test and Mann-Whitney test.

**Results:**

Among Cases, at least one significant stenosis (> 50% of the lumen) was detected in 348 occasions. Subsequent PTA was performed in 248 (71%). Median erythropoiesis-stimulating agent (ESA) weekly doses were higher in Cases than in Controls (8000 vs 5000 IU, *p* < 0.001). Cases received higher doses of intravenous iron/week than the Controls before the investigation (median 50 mg vs 25 mg, *p* = 0.004) and low molecular weight heparin (LMWH, *p* = 0.028). Compared to Controls, Cases had a lower level of parathyroid hormone (median 25 vs 20 ρmol/L, *p* = 0.009). In patients with diabetes mellitus, HbA1c was higher among Cases than Controls (50 vs 38 mmol/mol, *p* < 0.001). Multiple regression analysis revealed significant associations between Cases and female gender, prescription of doxazocin, and doses of ESA and LMWH.

There was no difference between the groups regarding hemoglobin, CRP or ferritin.

**Conclusion:**

In conclusion, the present study indicated that the factors associated with AVF problems were high doses of ESA, iron administration, and tendency of thromboembolism (indicated by high LMWH doses); the use of doxazocin prescription, however, requires further investigation.

## Background

A native arteriovenous fistula (AVF) is the preferred access for patients in chronic hemodialysis [1], while AV graft (AVG) and central dialysis catheters (CDC) are less preferred options [2]. Non-maturation of an AVF varies between 20% and 60% [3–6].

Although thrombosis, stenosis and infections appear as complications, the patency of AVF is superior to AVG and CDC [7–10]. The one-year patency of AVF is 50–70% [3, 11–13].

AVF dysfunction can be caused by too low bloodflow and/or clotting that develops predominantly upon stenosis due to neointimal proliferation [4]. The risk factors vary depending on the location of AVF dysfunction; for instance, AVF problems within the feeding artery are considered to be related to factors such as age, diabetes mellitus, hypertensive diseases, uraemia, tobacco use and inflammation [4, 6, 14]. Anastomosis related problems are considered mainly to be due to surgical measures [6]. Post-anastomosis related problems such as stenosis and thrombosis are noted especially in elderly patients (≥65 years), those with coagulation abnormalities, those with hypotension, and in smokers [6, 15, 16]. Other risk factors are prolonged clamping in conjunction with compression, repeated needle punctures and local hematoma [4, 6, 13, 17, 18].

Individual circumstances focus on the presence of diabetes mellitus, older age, and female gender, all of which are associated with a worse prognosis [14, 19–21]. Other factors that are considered to hamper AVF function are high levels of plasminogen activator inhibitor type 1, factor VII [22], daily dialysis [23], hypotension [24], smoking [25], disturbed calcium and phosphate balance and increased parathyroid hormone levels [26–29]. Previous studies from our center indicated that patients with AVF problems had received increased doses of erythropoietin stimulating agents (ESA) [30, 31]; this was also noticed in other studies [32–34]. However, further validation is needed to substantiate this.

Aside from individual suffering, the cost due to AVF complications are substantial [9, 35]. A patent AVF improves life expectancy [36]. Therefore, it is important to identify further variables that may be intervened to prevent AVF problems. In addition, if a CDC is necessary, this increases the risk for infections [9].

The aim of this study was to investigate if there are further risk variables that are associated with dysfunctional AVFs.

## Methods

### Patient characteristics

Since guidelines recommend lower arm AVF [37], the present study focused on risk factors for only these type of patients, thereby excluding those with AVG and upper arm placement of AVF, respectively. All patients were over 18 years of age. Data were collected between 2006 and 2015 from one university hospital and one county hospital. Specified data concerning non-maturation of AVF in the course of immediate surgical creation was not the intention of the present study since preoperative individual, surgical and technical evaluations differ. The intention was to clarify reasons for a failure after the AVF function had been established. Hemodialysis vintage (duration of time attending for dialysis), dialysis efficacy and present drug administration such as antihypertensives and anticoagulants are presented in Table 1.

**Table 1:** Characteristics of the study population at baseline presented as proportion, mean (SD) or median (quartiles)

Variable	Cases	Controls	***p***-value
	***n*** = 153	***n*** = 52	
Age, mean (SD)	65.9 (13.5)	65.7 (16.5)	0.565
BMI, mean	26.5 (4.9)	27.8 (6.1)	0.370
Hemodialysis vintage, mean, months	10.3 (33.9)	74.8 (45.1)	**< 0.001**
StKt/V, median	2.0 (1.4–2.4)	2.2 (2–2.4)	0.099
Female, n (%)	60 (39%)	13 (25%)	0.064
Tobacco:			
*Yes cigarettes, ever*	48 (46%)	37 (88%)	**< 0.001**
*Never*	57 (54%)	4 (10%)	**< 0.001**
*Yes snuff, ever*	0 (0%)	1 (2%)	0.6
*Missing data*	48 (31%)	11 (21%)	0.22
Diagnosis:			
*Glomerulonephritis*	25 (16%)	12 (28%)	0.28
*Diabetes Mellitus*	42 (28%)	10 (23%)	0.24
*TIN/Tubulointerstitial nephritis*	24 (16%)	3 (7%)	0.07
*PCKD/Polycystic kidney disease*	15 (10%)	3 (7%)	0.38
*Nephrosclerosis/Hypertension*	35 (23%)	11 (26%)	0.80
*Other*	11 (7%)	4 (9%)	0.90
Ongoing medication:			
*Calcium channel blockers*	61 (48%)	19 (43%)	0.608
*Alfa-1 receptor blockers*	40 (31%)	6 (14%)	**0.023**
*Furosemides*	79 (62%)	26 (59%)	0.758
*Beta-blockers*	94 (73%)	31 (70%)	0.757
*ACE-inhibitors*	34 (27%)	11 (25%)	0.839
*ARB:s*	56 (44%)	12 (27%)	0.054
*ASA*	69 (54%)	22 (50%)	0.654
*Vitamin-K antagonists*	9 (7%)	3 (7%)	0.962
*Statins*	68 (53%)	22 (50%)	0.720

*BMI*-Body Mass Index. *StKt/V* Standard Kt/V-weekly dialysis dose. *ASA* Acetylsalicylic acid. *ACE* Angiotensin Converting Enzyme. *ARB:s* Angiotensin II Receptor Blockers. Bold *p*-values indicate statistically significant values

### Study design

The study was an explorative retrospective observational study of 205 patients in chronic hemodialysis (HD) that had a native lower arm AVF for HD use. Of these, 153 patients were defined as ‘Cases’ since they had clinical assessed AVF dysfunction that resulted in referral for radiologic investigation at the local department of Radiology. The Cases performed a total of 473 radiologic examinations of AVFs. Each of these examinations was defined as an independent episode of access problems and as such was used for analysis. In addition to each radiological investigation (that could include/end up in intervention: radiological or surgical, Table 3), each episode included the closest regular laboratory samples within 1 month before and 1 month after (as cross-sectional data). Some patients underwent several episodes of interventions, so for these repeated laboratory data were obtained.

Fifty-two chronic HD patients who had not suffered any AVF complication (without need of radiology investigation) during at least 2 years (complication free period) served as Controls. Regular laboratory samples were collected within 2 months after the first and subsequent year respectively (complication free years). This study did not include any specific data of hyper- or hypotensive episodes during dialysis.

### Radiologic investigations and access flow measurements

For the Cases, the radiological investigations were based on clinical suspicion of insufficient AVF function. Referrals for radiological investigation were based on a combination of clinical findings, monitoring and surveillance measurements according to the KDOQI clinical practice guidelines [1]. The routines include a prospective trend analysis verifying a progressive impaired AVF access flow over time, especially if below 500 ml/min, as well as a repeated increase of static mean venous pressure by more than 50%, increasing recirculation, and/or insufficient cannulation possibilities. Often an ultrasound examination was also performed before radiological investigation to help guide stenosis diagnostics (Table 2). Results from radiological interventions and other measures were collected from medical records and from radiology archives (Table 3).

**Table 2:** The main reasons for radiological investigation (*n* = 473) of the AVF – usually confirmed by measured blood flow problems. The referrals included several surveillance and monitoring findings to discriminate possible expected clinical findings. “Others“ are a blend of worsened dialysis, pain, artery suction and others. Doppler Ultrasound was used as a preemptive examination in 307 of the investigations

Clinical finding	Total (%)
High venous pressure	36 (8)
Recirculation	31 (7)
Poor access flow	167 (35)
Local swelling of AVF area	19 (4)
Prolonged bleeding after decannulation	24 (5)
Cannulation problems	47 (10)
Preoperative investigation due to access problems	43 (9)
Others	94 (20)
Information missing	12 (2)

**Table 3:** The most common findings of radiological investigations (*n* = 473) and measures taken of Cases

Radiological findings:	Total (%)
Significant stenosis	348 (74)
No abnormality detected	40 (8)
Aneurysm	17 (4)
Other findings^a^	68 (14)
**Measures taken:**	
No direct radiological measure^b^	126 (27)
Additional radiological investigation	32 (7)
PTA-dilatation ± stent	248 (52)
Thrombolysis	14 (3)
Other	53 (11)

^a^ Collaterals, spasm, non-significant stenosis. ^b^ Of these, 27 were referred for surgical intervention, and the remaining 99 for subsequent PTA, coil or thrombectomy. *PTA* Percutaneous Transluminal Angioplasty

### Dialysis regimen

Access blood flow and recirculation (% of return of exit blood flow from the extracorporeal dialyzed circuit to the inlet blood flow) was measured using a Transonic® device. The total weekly administered doses of low molecular weight heparin (LMWH, U/week), the erythropoiesis-stimulating agents (ESA, U/week), and intravenously administered iron during the end of the HD (mg/week) were calculated. The values used were those within 1 month before and 1 month after the radiological investigation for Case and the one-year event free date for Controls. Dialysis efficacy was estimated by calculating the weekly stKt/V based on calculations of urea removal. The time spent in dialysis (hours/week) as well as body mass index (BMI) were also registered.

### Laboratory data

The laboratory values included were hemoglobin (reference 100–120 g/L), platelets (165–387 × 10^9^/L), serum values of albumin (g/L), calcium (2.10–2.50 mmol/L), phosphate (0.60–1.5 mmol/L), ferritin (7–120 μg/L), parathyroid hormone (1.5–7.6 ρmol/L), C-reactive protein (CRP,  < 5 mg/L, data < 5 mg/L are given as 4.99 mg/L), HbA1c (mmol/mol-IFCC), plasma fibrinogen (g/L), and cholesterol (3.9–7.8 mmol/L). Since we lacked information about weight in a number of patients, a rough estimate of ESA responsiveness/resistance (ESA/Hb) was calculated using the ESA doses (U/week) divided by the hemoglobin value (g/L). This was a modified estimate of ESA resistance based on the Erythropoietin Resistance Index by Santos et al. [38]. Similarly, the iron administration in mg/week was related to the hemoglobin level (Iron/Hb).

### Statistical analyses

Descriptive statistics were expressed as means with (SD = standard deviation) or as medians with (range). Analysis of categorical data was done with Chi 2 test, and group comparisons with respect to continuous variables were performed with Student t-test, ANOVA and Mann-Whitney test depending on data distribution. Paired statistics were performed with the non-parametric Wilcoxon rank test. When correlation analysis was performed the result was given as correlation coefficient ‘r’ for parametric (Pearson) and ‘rho’ for non-parametric (Spearman) analyses.

Statistical analysis was performed of the first episode and included in next step each new episode and new Control (one stenosis-free year later) as independent episodes. Baseline data in Table 1 are given for the first investigation of Cases and Controls.

The variables that differed between the two groups based on univariate comparisons (Table 4) were included in a multiple logistic regression. A stepwise logistic regression model was performed with group (Cases vs Controls) as the outcome including the variables gender, prescription of ESA, PTH, standard Kt/V (stKt/V), iron administration/week, low molecular weight heparin (LMWH) and alfa receptor-blockers. Several variables for HD-conditions were significant in univariate analyses, but due to strong correlations with each other we chose to include only stKt/v in the multiple model. Odds ratio (OR) and 95% confidence intervals (CI) together with R^2^ (coefficient of determination by Cox & Snell) are presented to quantify associations between the explanatory variables and the group as the outcome variable. A two-tailed significance level of *p* < 0.05 was used, if not otherwise mentioned. Statistical analyses were done with statistical package SPSS v25.

**Table 4:** Comparison (mean and SD) for Controls and for Cases. Data collected before vs after intervention (Cases) and for Controls (after 1 year free of AVF problems) and repeated within 8 weeks later for both groups. Also included are parametric and non-parametric comparisons between Controls first data and Cases before intervention

Variable	Controls				Cases				Group comparison	
	First data	Data < 8w later	n	***p***-value	Before intervention	After intervention	n	***p***-value	Parametric	Non-parametric
									Controls vs Cases	Controls vs Cases
									***p***-value	***p***-value
Calcium, mmol/L	2.34 (0.41)	2.35 (0.41)	117	0.674	2.35 (0.17)	2.35 (0.17)	417	0.736	0.729	0.737
Phosphate, mmol/L	1.60 (0.43)	1.57 (0.41)	117	0.548	1.65 (0.45)	1.66 (0.49)	413	0.873	0.193	0.214
Calcium x phosphate product	3.7 (1.0)	3.7 (1.0)	117	0.197	3.9 (1.1)	3.9 (1.2)	433	0.158	0.197	0.261
Albumin, g/L	36.9 (5.0)	37.4 (5.4)	118	0.107	36.5 (4.6)	36.2 (4.6)	407	0.052	0.308	0.432
Platelets, (×10^9^)	246 (88)	250 (95)	117	0.340	237 (76)	241 (82)	379	0.253	0.253	0.396
Hemoglobin, g/L	114 (12)	114 (12)	118	0.531	114.7 (11.9)	112.9 (12.3)	427	**0.001**	0.261	0.23
PTH, ρmol/L	33 (27)	34 (25)	100	0.740	27 (25)	34 (106)	349	0.253	**0.022**	**0.009**
Cholesterol mmol/L	3.89 (0.79)	3.78 (0.83)	10	0.547	4.2 (1.5)	3.8 (0.9)	30	0.169	0.231	0.524
HbA1c^a^, mmol/mol	49 (20)	54 (20)	17	**0.039**	58 (17)	58 (16)	101	0.448	**0.033**	**0.037**
Ferritin, μg/L	449 (244)	510 (489)	104	0.133	467 (255)	486 (251)	313	0.095	0.701	0.871
**Drug prescriptions:**										
ESA, U/W	4667 (3654)	4673 (3701)	78	0.975	7063 (387)	6982 (382)	333	0.153	**< 0.001**	**< 0.001**
Iron, mg/W	43 (65)	34 (35)	72	0.211	59 (60)	55 (44)	234	0.257	0.073	**0.004**
LMWH, U/W	11,949 (5062)	12,270 (5581)	74	0.282	14,225 (8022)	14,537 (8174)	227	0.183	0.064	**0.028**
**HD conditions:**										
Blood pump speed, ml/min	307 (28)	308 (20)	61	0.708	288 (48)	299 (46)	185	**0.001**	**< 0.001**	**< 0.001**
Recirculation, %	0.7 (4.5)	0.2 (1.7)	61	0.385	4.7 (11)	0.7 (3.7)	130	**< 0.001**	**< 0.001**	**< 0.001**
Access flow, ml /min	1105 (494)	1090 (497)	61	0.622	631 (446)	866 (512)	158	**< 0.001**	**< 0.001**	**< 0.001**
HD, hours/W	12.2 (2.7)	12.0 (2.3)	78	0.196	12.1 (2.4)	12.3 (2.4)	320	**0.009**	0.348	0.61
StKt/V	2.16 (0.53)	2.17 (0.58)	72	0.831	1.88 (0.59)	1.90 (0.59)	257	0.237	**< 0.001**	**< 0.001**
Venous pressure	137 (23)	140 (29)	62	0.492	133 (28)	131 (31)	121	0.409	0.263	0.295

*HbA1c results are given only for blood samples taken from patients with diabetes mellitus. *PTH* Parathyroid hormone, *ESA* Erythropoietin stimulating Agent, *LMWH* Low Molecular Weight Heparin, *HD* hemodialysis. Bold *p*-values indicate statistically significant values

## Results

A radio-cephalic (RC) fistula was present in 90% of the Cases, with a dominance for end-to-side anastomosis. The remaining 10% had a mid-arm placement. All Cases had been investigated by either percutaneous transluminal angiography (PTA) (mostly) or by phlebography due to impaired fistula function (Table 3). At least one significant stenosis (> 50%) was detected in 348 investigations, and a subsequent PTA was performed in 248 of these. Thrombolysis was performed in another 14 Cases (total *n* = 262). In 211 of the examinations, no radiological intervention was performed. In 40 of these examinations, there was no abnormality and the remainder underwent surgical adjustment or were referred for another more suitable radiological investigation/intervention (Table 3).

All Controls had a radio-cephalic (RC) fistula, with a dominance for end-to-side anastomosis. The 52 Control patients were considered for up to 124 measurements.

Table 1 shows that there were no differences between the groups in age, or between the prevalence of various diagnoses considered responsible for kidney failure. The Controls had a history of a longer vintage and more tobacco users. The Cases had a higher prevalence of administered alfa-receptor blockers (Table 1).

Table 4 shows that regarding baseline laboratory values the Cases had a higher dose of weekly iron administered and a lower parathyroid hormone (PTH) level than the Controls. However, the calcium-phosphate product did not differ between groups. For the patients suffering from diabetes nephropathy, the Cases had a higher HbA1c. This difference was not present after intervention. When comparing the first data for the Controls (*n* = 117) and the data before intervention in the Cases (*n* = 397), the median values of CRP did not differ (Controls 6 mg/L, quartiles 5–14 vs Cases 6 mg/L, quartiles 5–14).

Fibrinogen values were analyzed to estimate bleeding risk for 158 occasions before intervention in the Cases. The mean value was 4.5 g/L (SD ±1.3) and 70% had a value above the upper reference value.

The prescriptions differed insofar that the Cases received a higher dose of weekly ESA compared to the Controls (Table 4), iron and LMWH both before and after intervention.

The ESA doses did not differ between genders. The Cases with glomerulonephritis or nephrosclerosis received higher doses of ESA than those with other diagnoses (*p*-value< 0.01). In the Controls, the ESA doses were lower for all diagnoses compared to the Cases.

At follow-up the Controls with diabetes mellitus had an increase in HbA1c while other values remained unchanged. In the Cases, hemoglobin was lowered after intervention, most probably due to bleeding during the intervention. Indirect measures of the more effective AVF flow after intervention can be the increased blood pump speed, stKt/V and HD hours/week, and the decreased recirculation.

Among the Cases, the ESA/Hb correlated with fibrinogen (*r* = 0.292, *p* = 0.002), Iron/Hb (*r* = 0.215, *p* = 0.001), CRP (*r* = 0.163, *p* = 0.003), and dialysis time hours/week (*r* = 0.18, *p* = 0.001), but did not correlate with the other factors.

After initial analyses of the variables mentioned in the Statistical analyses section, proceeding univariate and multivariate logistic regression analyses are shown in Table 5. The results of the final model revealed that besides female gender (OR = 2.26, *p*-value = 0.012, R^2^ = 2%), a higher prescription of ESA (OR = 1.15, *p*-value< 0.001, R^2^ = 7%), the use of the alfa-receptor blockers doxazocin (OR = 3.99, *p*-value = 0.004, R^2^ = 4%, registered as a dichotomous variable), and higher doses of LMWH (OR = 1.06, *p*-value = 0.013, R^2^ = 2%) remained as statistically significant factors for the performance of AVF investigation.

**Table 5:** Logistic regression model with group (Case = 1 and Control = 0) as the outcome variable (Odds ratio -OR- and 95% confidence interval- CI). Variables associated with group are investigated by univariate and multivariate models

	Univariate		Multivariate model			
			Step I ^**a**^		Final model ^**b**^	
Variable	OR (CI)	*p*-value	OR (CI)	*p*-value	OR (CI)	*p*-value
Iron, mg/W	1.01 (1.00–1.02)	0.002	1.01 (0.99–1.01)	0.209	–	–
ESA, U/W^d^	1.11 (1.06–1.17)	< 0.001	1.15 (1.07–1.23)	< 0.001	1.15 (1.07–1.23)	< 0.001
LMWH, U/W^d^	1.04 (0.99–1.08)	0.054	1.06 (1.003–1.11)	0.049	1.06 (1.01–1.11)	0.013
Age	0.99 (0.98–1.01)	0.874	–	–	–	–
Female	2.35 (1.47–3.76)	< 0.001	1.91 (0.96–3.80)	0.063	2.26 (1.19–4.27)	0.012
StKt/V	0.40 (0.23–0.61)	< 0.001	0.59 (0.28–1.22)	0.158	–	–
Doxazocin	3.07 (1.58–5.95)	0.001	2.74 (1.04–7.24)	0.042	3.99 (1.57–10.13)	0.004
CRP	0.99 (0.99–1.07)	0.545	–	–	–	–
PTH	0.99 (0.98–0.99)	0.027	0.99 (0.98–1.01)	0.823	–	–
HbA1c ^c^	1.04 (1.02–1.07)	< 0.001	-		–	–

^a^Variables from the univariate analysis with *p*-value< 0.1 included in step I; ^b^Backward Stepwise model (Wald), ^c^HbA1c included several missing data (around 50%) and is therefore not included in the multivariate model, ^d^OR calculated for a change of a thousand units. HD condition variables were strongly correlated to each other, so we chose to include only one - StKt/V

Iron administration dropped out from the final model eventually depending on correlation to ESA and/or LMWH (iron vs ESA r = 0.252, *p* < 0.001; iron vs LMWH r = 0.139, *p* < 0.05). In patients with diabetes mellitus, there was an inverse correlation between the HbA1c level and extent of dialysis, measured as stKt/V (rho = − 0.242, *p* = 0.017, *n* = 97). Doxazocin was not prescribed as the sole antihypertensive medication in any patient but was prescribed together with two to five other antihypertensives in 93% of the patients. In those with doxazocin, stKt/V was lower than for patients without such prescription (*p* < 0.001), but did not differ between Cases and Controls. In patients without doxazocin, stKt/V was lower in Cases before radiological investigation than in Controls (*p* = 0.028).

## Discussion

The complexity of AVF dysfunction is due to multiple reasons [14, 18]. It appears different on various levels of the AVF such as the arterial branch, the area of the anastomosis, and the area of cannulation. In the present study, with the Case and Control design, several new plausible risk factors were found. After multiple regression analyses, the remaining risk factors for AVF dysfunction, besides female gender, were the prescriptions of higher doses of ESA and LMWH and the use of alfa-receptor blockers. Univariate analyses showed several significant findings but with limited r-square values, which highlights the complexity of numerous different reasons that contribute to AVF morbidity. One reason might be the relation to the prescription of higher doses of iron for the Cases. Although there was no difference in CRP, ferritin and albumin during the first and the second episode (after investigation), there was a small and significant correlation between ESA/Hb and CRP. An inflammatory association between AVF problems and CRP was also shown by others [39]. However, we cannot rule out that the CRP may be a primary reason caused, for example, by the poorer general condition of these patients, but in contrast may also be a consequence of AVF dysfunction. Elevated ESA doses in the Cases with AVF dysfunction were also shown in a few previous studies [30, 31, 40, 41].

The other deviate findings in the Cases are reasonably explained by the impaired blood flow in the AVF before intervention such as the use of a lower blood pump speed, higher recirculation, less effective dialysis (shown by a lower stKt/V), and the shortened/interrupted HD sessions. All these variables improved after intervention. Within the frame of the study, other expected risk factors such as tobacco use and calcium, phosphate and PTH levels were similar or even worse in the Controls.

Insulin resistance due to tissue insensitivity is pronounced in uremic patients [42]. In the present study, an inverse correlation between HbA1c and stKt/V in patients with diabetes mellitus indicate that less dialysis is associated with a worse metabolic control. Such metabolic disturbance may impair the vascular endothelium [11, 43]. This is also in line with the present study where the Cases with diabetic nephropathy had higher HbA1c values before intervention, which thereafter improved and became similar to Controls.

However, the shorter vintage when AVF problems appear in the Cases compared to the Controls may be due to the maturation process, although this period is mainly considered to be within 8 weeks after surgery [1]. Therefore, these results indicate that the Cases mainly have a more general problem that affects their AVF already early on, but also later after placement.

Several Cases in the present study might suffer from hypo-responsiveness to ESA since hemoglobin levels were similar to Controls. Johnson et al. noted that such hypo-responsiveness is present in 5–10% of chronic kidney disease patients [44]. A few studies have suggested a link between ESA responsiveness and higher morbidity and mortality in end-stage renal disease patients [45, 46], often together with more signs of inflammation [47–49]. Regarding AVF in the present study, there is no indication that the Cases had a higher degree of inflammation than the Controls. In that ESA increases blood pressure [50], higher ESA doses could explain the addition of antihypertensive drugs such as alfa-receptor blockers.

The activation of coagulation during HD is initiated by the blood membrane contact [51]. This will result in high concentrations of activators in the blood that return to the AVF, which may favor clotting in this AVF area. Besides a local activation, a more general effect seems present since most AVF stenoses and thromboses are present/develop closer to the AVF anastomosis as well as the site where the needle for the inlet to the dialysis circuit is located [14]. Other studies have shown that a higher ESA dose was related to a higher extent of thromboembolic complications in AVF [52] as well as of cardiovascular diseases in general [34, 52–54].

The present study shows a relation between ESA/Hb and plasma fibrinogen that indicates an association with increased thromboembolic risk. Such data also fit with previous studies that noted in general that treatment with ESA was associated with increased thromboembolic events [32–34]. Inflammation may induce increased fibrinogen levels. However, in the present study, the CRP values, as a marker for inflammation, did not differ extensively between the Cases and Controls, and in general were not very high.

The increased LMWH doses in Cases versus Controls may well indicate that LMWH is a confounding factor for an increased tendency for clotting that is visible in the dialyzer and/or in the venous air traps. The increasing number of extracorporeal circuit thromboses oblige the dialysis staff to increase the dose of LMWH. Such thromboembolic tendency in these patients may increase the risk also for thromboses associated to the AVF.

Another possible consequence of increased LMWH doses may be increased blood loss after decannulation at the site of the AVF after termination of the HD. The blood loss would explain that higher doses of ESA and intravenous iron were used in these patients. The blood loss would also lower iron stores, increase the need for iron supplementation and explain that ferritin levels were similar to those of the Controls. However, the repeated infusions of iron could have toxic effects on local AVF conditions and lead to negative effects on all-cause mortality [55, 56]. Also, ferritin levels above 400 μg/L have recently raised concern to be associated with higher inflammatory effects on the vascular endothelium with iron-induced oxidative stress and endothelial dysfunction [57, 58]. In the present study, ferritin was above 400 μg/L among half of the patients. However, this level was recommended by KDIGO in 2012 [59], which indicates that lower values may be considered in the future. Maintaining a regular hemoglobin level can be achieved by using regular ESA doses combined with intravenous iron doses adapted to S-ferritin (SF) and transferrin saturation (TSAT) thresholds that are lower than those used in routine practice; this contributes to a reduced risk of iron overload [60].

Studies have indicated increased morbidity and shortened life expectancy for HD patients that receive ESA above 8100  U/week [61], and iron doses above 800 mg within 6 months [62]. The administration of intravenous ESA might enhance erythropoietin receptors in the stenotic fistulae, thus inducing cell proliferation in response to local TGF-β1 expression, and result in intimal thickening of the vessel wall [63]. This should entail more careful attention when reaching doses beyond 8000 U/week and subsequent attention to other methods to treat the anemic state of the HD patient.

The lower stKt/V, as an estimate of the extent of HD, was still significantly lower for the Cases (compared to the Controls) after intervention of the stenosis, although it was well within the limit of recommendation. Therefore, the present study cannot rule out that extended HD could reduce the need of ESA, which would be in line with other studies [64, 65].

The significant finding of a higher frequency of doxazocin prescription together with other antihypertensive drugs may imply that these patients suffered from a more resistant hypertension. Such hypertension is not evidently related to interdialytic weight gain [66]. This could be related to higher doses of ESA that may increase blood pressure [50]. On the one hand, doxazocin dilates vessels and may counteract, for example, platelet aggregation, thereby having beneficial profibrinolytic effects [67, 68] and counteracting coronary constriction [69]. On the other hand, doxazocin may stimulate collagen synthesis [70] and induce edema [71], which would be a disadvantage for the AVF. We recommend further studies to clarify if advantages or disadvantages by doxazocin and AVF function exists.

The strength of the present study was the consecutive inclusion of the numerous episodes of the Cases and the Controls who had a lower arm AVF observed up to a 9-year period.

### Limitations

Repeated use of Cases and Controls may be a limitation. The controls had a wash-out period of 1 year and were considered as new controls for the next episode the following year. In some Cases, repeated measurements were performed several times and we considered each episode as independent. Within both groups paired statistics were used with each patient as his/her control in the analysis (before vs after for each episode). Therefore, results on both the group and individual level are presented. Variables such as gender and diagnosis were stable over time. Age alterations were considered similar in both groups (see Table 1)*.*

Information about overhydration between dialysis and ultrafiltration was not included in the analyses. This is a known factor for hyper- and hypotension and AVF access [72]. In some of the patients, the phlebography or angiography could not reveal a stenosis, although the AVF access was not considered optimal from a clinical point of view. This could have been due to vasoconstriction or problems to achieve adequate locations of the needles at the insertion sites. On some occasions, it was later found that a patient had more central stenoses such as in the feeding artery or in veins of the upper arm or at the exit of the subclavian vein. Some patients suffered from elongated ‘non-significant’ stenoses that caused an impaired flow [14]. We cannot rule out that hypotensive episodes appeared in patients that were prescribed the antihypertensive drug doxazocin. However, hypotension is probably less frequent in those patients prescribed numerous antihypertensives due to therapy resistance than in those without hypertension.

The importance of the results is that the presence of hypo-responsiveness to ESA and iron doses should bring into question whether it might be reasonable to keep a lower dose and thereby a hemoglobin level in the lower range. In patients with previous clotting problems, complicated surgical procedures and higher fibrinogen levels favor thromboembolic events, and therefore may be considered for a prolonged period of LMWH as prophylaxis for thrombosis after AVF surgery, as is also suggested by others [73]. In the present study, the relation between ESA/Hb and plasma fibrinogen indicates an association and supports an increased thromboembolic risk. Such data also fit with previous studies showing that treatment with ESA was associated with increased thromboembolic events [32–34].

Future research should clarify if a more extensive bleeding after decannulation, caused by higher LMWH doses contributes to anemia and increased doses of ESA.

Diabetes patients may benefit from an improved metabolic control to counteract insulin resistance. Combining high doses of ESA and intravenous iron among diabetics treated with HD may contribute to AVF related complications as is shown by other researchers [58]. The present study does not rule out that a similar approach may also be helpful for other groups of patients who suffer from AVF problems.

In conclusion, the present study indicated that the factors associated with AVF problems were high doses of ESA, iron administration, and tendency of thromboembolism (indicated by high LMWH doses); the use of doxazocin prescription, however, requires further investigation.

## Data Availability

The datasets used and/or analyzed during the current study are available from the corresponding author on reasonable request.
